# Recent Advances in Carbon-Based Catalysts for Heterogeneous Asymmetric Catalysis

**DOI:** 10.3390/molecules30122643

**Published:** 2025-06-18

**Authors:** Yidan Zheng, Tianze Liu, Jingyou Tai, Ning Ma

**Affiliations:** Department of Chemistry, School of Science, Tianjin University, Tianjin 300072, China; zhengyidan@tju.edu.cn (Y.Z.); chem_liutianze@tju.edu.cn (T.L.); taijingyou@tju.edu.cn (J.T.)

**Keywords:** carbon material, graphene, carbon nanotube, asymmetric catalysis

## Abstract

Carbon materials, including graphene, carbon nanotubes, and fullerenes, serve as effective supports for catalysts and play a pivotal role in heterogeneous asymmetric catalysis due to their unique properties and ability to create defined environments for catalytic reactions. Recent research has focused on developing novel carbon-based catalysts that combine the advantages of heterogeneous catalysis with enhanced stability and reusability. This review highlights the synthesis and catalytic applications of graphene, carbon nanotubes, and fullerenes as heterogeneous support materials in asymmetric organocatalytic and organometallic reactions, covering their mechanisms, efficiency, and potential for advancing sustainable chemical processes.

## 1. Introduction

Pioneering research work from Knowles, Noyori, Sharpless, List, and MacMillan has enlightened the asymmetric synthesis of diverse chemical products in a greener manner, substantially improving efficiency and avoiding waste [1,2]. Nevertheless, these homogeneous asymmetric catalysts were generally soluble in the reaction mixture, which made them challenging to recover and reuse due to the difficulty of separation from the reaction mixture. To successfully recycle the catalysts, heterogeneous asymmetric catalysts have been developed. In the early stage, supports such as silica [1], alumina, and polymers [2,3] have been focused on to immobilize metal-based catalysts. These developments increase the efficiency and sustainability of catalytic processes [4], but challenges remain in optimizing the enantioselectivity of these systems [1,5,6].

Nowadays, researchers are very concerned with carbon materials because of their excellent characteristics and unique applications in heterogeneous systems. Carbon-based materials, especially nanocarbon materials, including graphene, carbon nanotubes (CNTs), and fullerenes (C60), have increasingly been involved in the development of heterogeneous catalytic systems [7]. These materials are capable of serving as effective supports in heterogeneous catalytic systems, due to their high chemical stability, good thermal conductivity, and strong mechanical resistance with a lightweight nature. Additionally, they can be functionalized with catalytically active moieties, which allows for the construction of heterogeneous catalytic systems by immobilizing homogeneous catalysts, thereby enhancing their suitability in a wide range of reactions.

In homogeneous asymmetric catalysis, soluble asymmetric catalysts are hard to recover and lack reusability [4]. The immobilization of catalytic scaffolds onto the surface of support materials is a powerful strategy, which usually distributes the catalytic sites crucial for enantioselective catalysis into the pores on the surface areas. However, there are still some challenges in nanocarbon material immobilization, such as the pore blocking phenomenon, which could cause the undesired decrease in efficiency and selectivity of catalytic processes. Furthermore, traditional homogeneous catalysts are soluble in the reaction mixture, and their corresponding pollutants can result in long-term ecological damage, especially when they contain heavy metals or other hazardous materials. In contrast, heterogeneous catalysis using carbon-based materials offers significant advantages in terms of sustainability. Carbon materials, being inherently reusable and recoverable, provide an eco-friendlier alternative to homogeneous catalysts. These catalysts can be easily separated from the reaction mixture, reducing waste and pollutants.

Graphene is a single layer of sp^2^-bonded carbon arranged in a honeycomb lattice, which provides a huge surface area, enabling a fairly strong interaction between catalytic areas and reactants. In most catalytic usage, pristine graphene does not act as the main character; instead, its derivatives, such as graphene oxide (GO) and reduced graphene oxide (rGO), are favored due to their high modifiability, which arises from the presence of heteroatomic groups on its derivatives, allowing for the introduction of catalytic active groups. Recently, graphene derivative-based catalysts have been involved in various asymmetric reactions, such as C-C bond formation and hydrogenation reactions.

Carbon nanotubes are composed of single-layer graphene sheets rolled into cylindrical shapes and can be categorized into two main types—single-walled CNTs and multiwalled CNTs. CNTs have a high surface area, excellent thermal and chemical stability, and high conductivity, which endow them with great application potential. Various strategies for using CNTs as effective supports have been developed, such as the covalent immobilization of catalytic moieties and noncovalent loading of metal catalyst.

In several previous reviews, researchers have concentrated on the catalytic usage of carbon materials, especially graphene-based materials [8,9,10,11,12]; however, there is lack of a comprehensive review about carbon-based asymmetric catalysis. The aim of this review is to introduce recent advances in the application of carbon-based materials for heterogeneous asymmetric catalysis. The synthetic strategies, catalytic performance, and promising results from recent studies are summarized. By focusing on the types of catalysts immobilized on the carbon supports, including organocatalysts, metal nano particles, and metal–organic complexes, this review presents a comprehensive understanding of how carbon materials effected the performance of heterogeneous asymmetric catalysts in the recent representative examples.

## 2. CNTs Based Asymmetric Catalysts

CNTs have emerged as promising supports for catalysts due to their unique properties, including high surface area, chemical stability, and ease of functionalization [13,14,15]. The integration of CNTs with asymmetric catalysts has opened a new avenue in asymmetric catalysis, allowing for the development of heterogeneous catalysts that combine the advantages of high activity and selectivity, ease of separation, and recyclability. Organocatalysts, metal nanoparticles, and metal–organic complexes are immobilized on CNTs as the main types of asymmetric catalysts. In this section, we summarize their applications along these lines.

### 2.1. CNTs Based Organocatalysts

Chiral organocatalysts were generally introduced on CNTs mainly via two distinct methods in the recent reports. The first is covalent interactions, while the second is featured with supramolecular assembly.

**Scheme 1:**
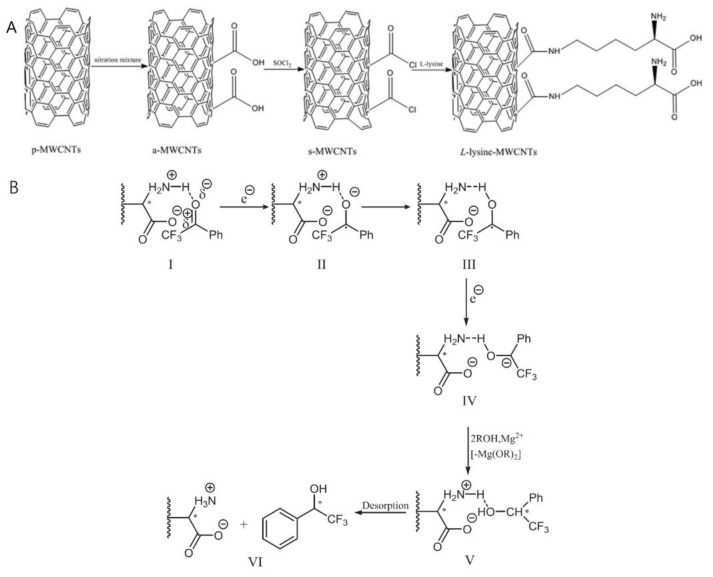
The process of *L*-lysine-MWCNT synthesis and the proposed pathways of asymmetric catalysis reaction. * Represents chiral carbon. Reproduced with permission from [16].

By covalent bonding, Lu et al. prepared chiral amino acids functionalized CNTs [16], which opened new avenues for the development of metal-free chiral catalysts. These catalysts, prepared by grafting *L*-lysine onto the surface of multi-walled carbon nanotubes (MWCNTs) as shown in Scheme 1, have demonstrated remarkable efficiency in the asymmetric electroreduction of aromatic ketones. The resulting amino acid-functionalized MWCNTs not only exhibited good catalytic performance but also showed excellent stability and reusability, making them attractive candidates for sustainable catalytic processes. However, the primary disadvantage of this type of catalyst is its low enantiomeric excess (ee) of (*R*)-products, which is no greater than 45%.

**Scheme 2:**
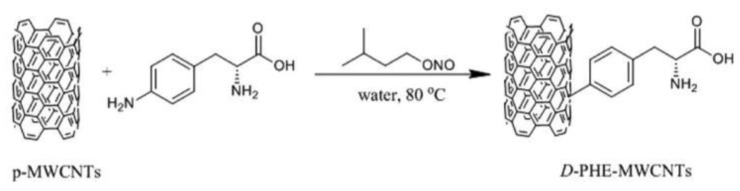
The process of *D*-PHE-MWCNT synthesis. Reproduced with permission from [17].

Building on this concept, the use of different amino acids for the functionalization of CNTs has been explored in further research. As highlighted in the work by Lu et al. [17], the choice of amino acid is crucial for the success of the asymmetric catalysis. When D-phenylalanine was grafted onto MWCNTs, as shown in Scheme 2, (*S*)-products have been provided with high ee values, indicating the importance of the chiral environment in these transformations.

**Figure 1:**
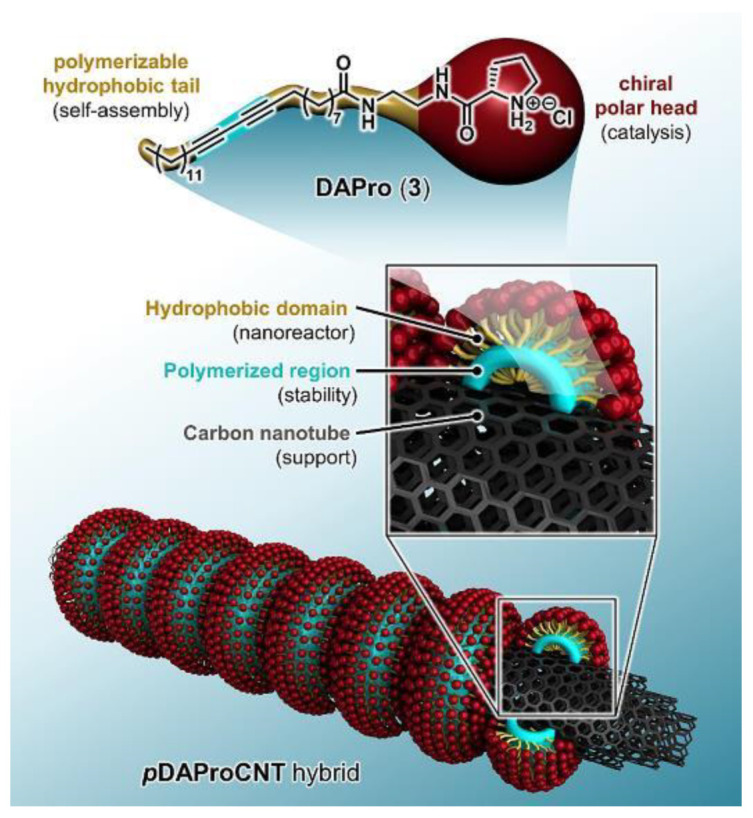
Schematic illustration of pDAProCNT hybrid structure. Reproduced with permission from [18].

Nevertheless, the direct grafting of chiral amino acids onto CNTs presents a limitation—the relatively low ee observed in these systems. The innovative approach of using supramolecular assembly for the immobilization of proline amphiphiles on CNTs described by Doris et al. [18], as shown in Figure 1, represents a significant advancement in the field. This method allows for the creation of a hydrophobic domain at the CNT interface, which accommodates hydrophobic reactants and stabilizes the transition state, leading to improved enantioselectivity and ee varied from 46% to 98%. The resulting nanohybrid catalysts have been successfully employed in asymmetric aldol reactions in water, thereby demonstrating the potential of CNT-based organocatalysts in sustainable chemistry.

### 2.2. CNTs Based Metal Nanoparticles

The unique electronic properties, chemical stability, and large surface area of carbon nanotubes have been demonstrated to make them highly useful for supporting metal nanoparticles [19].

Zhou et al. [20] described the earliest instance of the utilization of Pt nanoparticles based on CNTs for asymmetric catalysis. In this work, they prepared a type of Pt deposited carboxylated CNTs. The catalyst was then used to catalyze the asymmetric hydrogenation of ethyl pyruvate in the presence of (-)-cinchonidine. The significance of this work lies in the successful demonstration that Pt nanoparticles can be stably deposited on CNTs and used for asymmetric catalysis. However, the enantioselectivity of this reaction is relatively low.

Based on this research, Li et al. [21,22] further optimized the deposition of metal nanoparticles and encapsulated Pt nanoparticles in CNTs. The confinement effect within the CNT channels was found to enhance the performance of the Pt nanocatalyst, with a turnover frequency (TOF) above 1.0 × 10^5^/h and ee up to 96%. This high activity was attributed to the ultrahigh enrichment of the chiral modifier and reactants inside the CNT channels, as revealed by transmission electron microscopy (TEM) analysis and adsorption studies. Furthermore, other chemists optimized the catalytic systems, achieving ee up to 99% and enabling good reusability of the catalysts [23].

**Figure 2:**
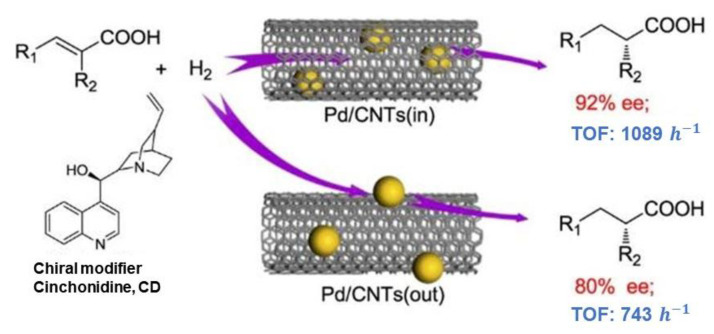
The difference in enantioselectivity due to Pd nanoparticle localization in CNTs. Reproduced with permission from [24].

The application of CNTs in asymmetric catalysis has been further expanded to include Pd nanoparticles for the enantioselective hydrogenation of α,β-unsaturated carboxylic acids [24]. Pd nanoparticles confined within CNTs exhibited higher activity and enantioselectivity compared to those located outside the channels, with enantioselectivity as high as 92%, as shown in Figure 2. This work underscores the importance of the CNT channels in enriching reactants and chiral modifiers, thereby enhancing the catalytic performance.

### 2.3. CNTs Based Metal–Organic Complexes

The integration of metal–organic complexes onto CNTs can be achieved through both covalent and noncovalent functionalization, each offering distinct advantages. Covalent functionalization provides a robust linkage between the metal–organic complexes and the CNTs surface, ensuring the stability and reusability of the catalyst. This approach often involves the modification of CNTs with functional groups such as carboxyl, hydroxyl, or amine groups, which can then react with the metal–organic complexes to form stable bonds [25,26]. Noncovalent functionalization, on the other hand, relies on interactions such as π-π stacking [27,28], van der Waals force [29], and self-assembling [30,31,32,33,34,35] to immobilize the metal–organic complexes onto the CNTs surface. This method preserves the native properties of the CNTs and the metal–organic complexes, often leading to catalysts that exhibit high activity and selectivity.

The first illustration of a CNT-based metal–organic complex chiral catalyst was presented by Baleizão et al. (2004) [25]. A type of functionalized CNT was successfully produced and used to catalyze the cyanosilylation of aldehydes with trimethysilylcyanide by introducing sulfhydryl groups onto the CNTs, followed by the attachment of vanadyl salen complexes. However, the enantiomeric excess obtained with the as-prepared CNT-based vanadyl salen complex catalyst is significantly lower than those using related homogeneous catalysts in solution (up to 94%) [36].

**Scheme 3:**
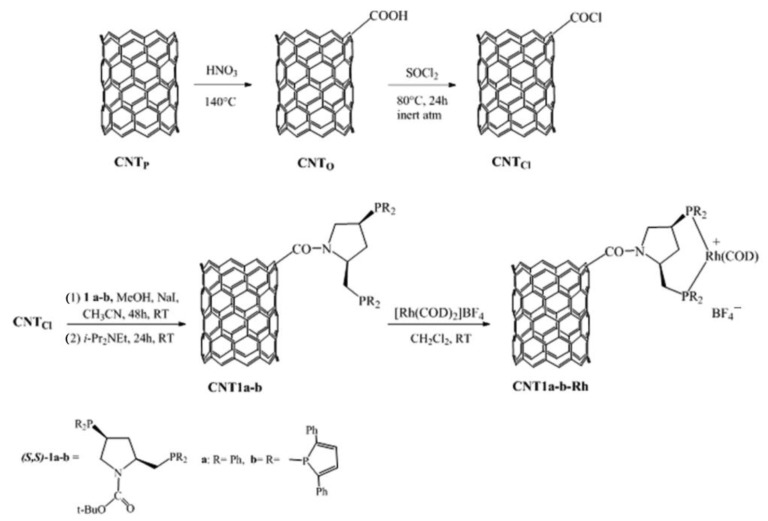
The process of CNT1a-b-Rh synthesis. Reproduced with permission from [26].

**Scheme 4:**
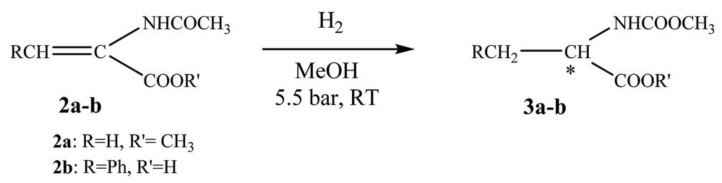
The asymmetric reaction between methyl 2-acetamidoacrylate and α-acetamidocinnamic acid. * Represents chiral carbon. Reproduced with permission from [26].

Then, chiral rhodium complexes were covalently immobilized on CNTs for enantioselective hydrogenation by Román-Martínez and coworkers [26], as shown in Scheme 3. In this work, the CNT-based chiral rhodium complex has been used to catalyze the asymmetric hydrogenation of methyl 2-acetamidoacrylate and α-acetamidocinnamic acid, as shown in Scheme 4. The highlight of this work is that the catalyst can be recycled three times and still maintain a conversion of 100%. Furthermore, the stereoselectivity of the catalytic products can be attributed to the steric effect of the ligands.

In addition to covalent bonding, the noncovalent functionalization of CNTs has also attracted widespread interest. Zhou et al. firstly explored an approach for the noncovalent functionalization of CNTs [27]. In this work, a chiral metal complex was synthesized by combining a pyrene derivative with a Pyrphos ligand and Rh(I). Then, this kind of rhodium catalyst was absorbed by CNTs via π-π stacking interaction between the pyrene and CNTs. In the asymmetric hydrogenation of α-dehydroamino esters, this catalyst demonstrated excellent enantioselectivity, reaching up to 96% ee, and exhibited remarkable stability, retaining 92% ee after 10 recycling cycles.

**Figure 3:**
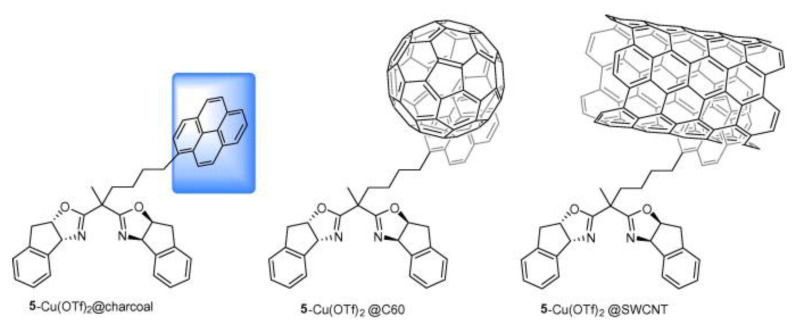
The structure of metal complexes immobilized on difference carbon materials. The blue square indicates the region interacting with charcoal through π–π stacking. Reproduced with permission from [28].

**Scheme 5:**
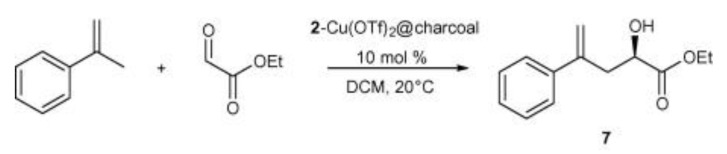
The ene reaction between ethyl glyoxylate and α-methylstyrene. Reproduced with permission from [28].

In a similar approach, Schulz et al. achieved asymmetric catalysis for the Henry reaction and ene reaction by changing the ligand and the metal [28]. In this work, two kinds of metal complexes were prepared by combining bis(oxazoline) ligand with anthracene and pyrene, respectively. Then, they were immobilized on charcoal, fullerene, and CNTs through π-π stacking, as shown in Figure 3. The ene reaction between ethyl glyoxylate and α-methylstyrene was conducted using a CNT-based catalyst, as shown in Scheme 5, achieving 64% ee and remaining at 62% after seven recycling cycles.

In another work, Kobayashi et al. initially took advantage of the strong van der Waals force between CNTs and dodecylsulfate to immobilize a kind of Lewis acid, zinc(II) ions, onto the surfaces of CNTs without any similar perturbation of the CNTs structure in covalent modification [29]. In this work, the hydrophobic and electronic properties of CNTs were combinedly considered. CNTs provide a large hydrophobic surface area, which facilitates the adsorption and enrichment of hydrophobic reactants and ligand molecules, thereby enhancing reactivity and selectivity. Furthermore, the incorporation of surfactants into the system facilitates the dispersion of CNTs in water, thereby ensuring the sustained dispersion of nickel catalysts within the system. Besides, the presence of electronic interactions between CNTs and nickel catalysts changes the electronic properties of nickel catalysts, which in turn improves catalytic performance. As-prepared CNT-based nickel catalyst was used to catalyze the enantioselective conjugate addition of benzaldoxime in water with ee up to 95%.

Shibasaki et al. have explored a novel kind of immobilization methodology of metal–organic complex on CNTs and reported various results on this approach [30,31,32,34]. This kind of chiral catalyst was prepared via self-assembling neodymium, sodium, and a ligand based on CNTs [30]. The catalyst displayed enhanced catalytic efficiency for the Henry reaction and could be readily reused through straightforward filtration. Comparative experiments have shown that restricting self-assembled asymmetric catalysts on CNTs can significantly improve their catalytic efficiency and enantioselectivity, reaching up to 95% ee. Due to the fact that by restricting the self-assembly process, the smaller and more dispersed catalyst clusters were produced, thus increasing the specific surface area and active sites of the catalysts.

Then, some improved preparation procedures and full details of this kind of Nd/Na heterobimetallic–organic complex were studied [31]. In the subsequent work, researchers decreased the amount of chiral ligand, improved the loading ratio of the catalyst components, and explored the effect of pretreatment on the reaction to achieve higher catalytic efficiency and enantioselectivity. Furthermore, the group discussed several promising intermediates of natural products or drug candidates that could be synthesized using the newly prepared catalyst.

**Figure 4:**
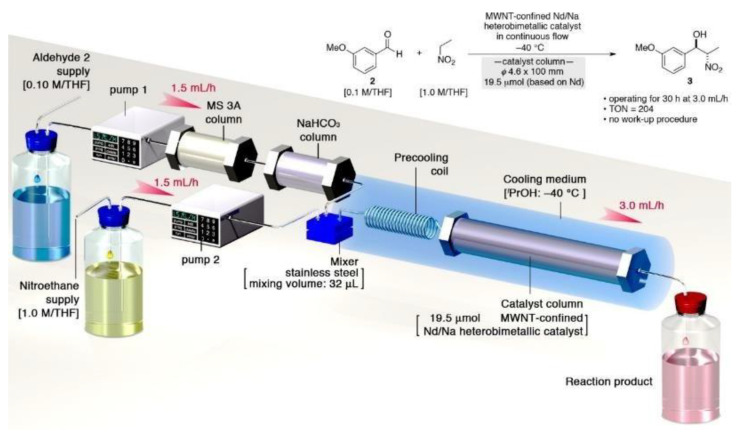
The scheme of the Henry reaction in the flow system. Adapted with permission from [32].

In a subsequent study, the researchers constructed a continuous flow platform for asymmetric catalysis of Henry reactions [32], as shown in Figure 4. The platform’s key features include the use of self-assembled CNT-based Nd/Na heterogeneous catalysts, which were readily prepared. The flow system permitted the continuous production of the desired chirality-enriched trans-1,2-amino alcohol product, while the quenching operation was obviated and the cooling system was minimized. The catalyst demonstrated high activity and stereoselectivity, enabling the scalable synthesis of over 10 g of the target compound. This work illustrates the value of integrating heterogeneous asymmetric catalysts into a continuous process for the efficient production of valuable synthetic intermediates.

In addition, this team put forth a novel approach for the synthesis of Nd/Na heterometallic catalysts, utilizing the more stable, cost-effective, and readily accessible NdCl_3_·6H_2_O instead of the previously employed NdO_1/5_(O*^i^*Pr)_13/5_ [34]. The new catalysts were prepared by self-assembling NdCl_3_·6H_2_O through the mixing of the compound with NaO*^t^*Bu in specific ratios. This method simplifies the preparation process and reduces the cost, as it can be performed at room temperature without the need to operate in a glove box. As such, this approach refines the continuous flow reaction platform and demonstrates the potential of this catalyst for industrial applications.

In another work of this team, the metal Nd was replaced by Er, and a kind of asymmetric Mannich-type reaction catalyst was prepared [33]. In this work, Er(O^i^Pr)_3_ was used to obtain the heterogeneous catalyst based on CNTs through the preparation methodology analogous to that of the Nd/Na heterobimetallic–organic catalyst [30]. The so-obtained Er catalyst was employed to catalyze the Mannich-type reaction to achieve a high stereoselectivity (ee up to 98%).

## 3. Graphene-Based Catalysts

As a single-layer, two-dimensional sheet of graphite tightly bounded by sp^2^-hybridized carbons arranged in a honeycomb lattice, graphene is endowed with exceptional strength, lightness, and flexibility properties due to its unique structure. However, the graphene derivatives, such as graphene oxide (GO), are preferred in most catalytic usage. In contrast to pristine graphene, GO serving as a catalyst support offers several advantages, such as affordability and ease of surface modification. The catalytic active species are commonly immobilized on GO via the following two different ways: (1) noncovalent functionalization thought π-π stacking or other interactions such as hydrogen-bonding and/or ionic interaction and (2) covalent functionalization. In recent years, GO proved to be an excellent support for chiral catalysts in several organic reactions, such as Michael addition [37], aldol addition [38,39], and other reactions [40].

### 3.1. Graphene-Based Organocatalysts

**Scheme 6:**
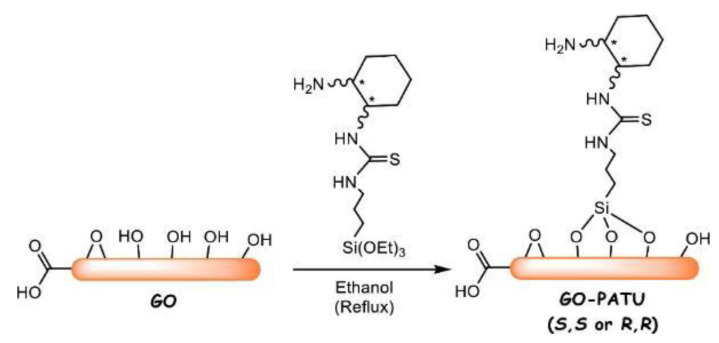
The process of GO-PATU synthesis. * Represents chiral carbon. Reproduced with permission from [37].

Enantioselective Michael addition is of great importance for synthesizing pharmaceutical intermediates. Durmaz et al. prepared chiral amine–thiourea organosilane and covalently bonded it onto GO via silane coupling reaction to prepare (*S*,*S*)- and (*R*,*R*)-GO-PATU as new bifunctional catalysts [37], as shown in Scheme 6, which have excellent catalytic performance in enantioselective Michael addition. The catalysts exhibited high stereoselectivity in the addition of isobutyraldehyde to trans-β-nitrostyrene with excellent reusability over multiple cycles. The best results were achieved under room temperature using dichloromethane as the solvent and D-(or L-) camphorsulfonic acid as an additive. The enantiomeric excess (ee) values of addition products from various substrates ranged from 77% to 95% with high yields (58–85%).

**Scheme 7:**
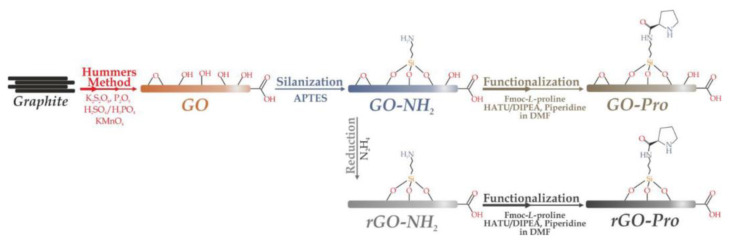
The process of GO-Pro and rGO-Pro synthesis. Reproduced with permission from [39].

Since the L-proline was reported as an effective asymmetric catalyst for aldol reaction in 2000s, the research of its immobilization has been continuously evolving to enhance the reusability and avoid environment problems. Bingol et al. reported a simple way for anchoring L-proline onto the GO/reduced-GO (rGO) via covalent bonding to produce a new asymmetric catalyst GO-Pro/rGO-Pro for aldol reaction [39]. In this method, GO-involved silanization with aminopropyltriethoxysilane (APTES) introduces thevamine group onto the surface of GO, creating a reactive site to anchoring L-proline, as shown in Scheme 7. The GO-Pro and rGO-Pro were used to catalyze direct asymmetric aldol reaction in hexane, achieving up to 88% isolated yield and 85% ee with the aid of benzoic acid. Notedly, the catalyst retained high efficiency over four cycles.

**Scheme 8:**
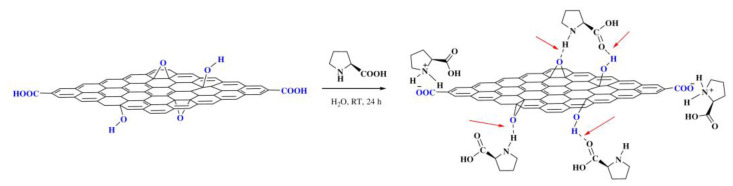
The noncovalent immobilization strategy of anchoring L-proline. The red arrows demonstrate hydrogen bonds. Reproduced with permission from [38].

Yin et al. employed a different strategy anchoring L-proline onto GO via noncovalent method, where L-proline was loaded through hydrogen bonding and ionic interactions to provide L-proline/GO hybrid catalyst [38], as shown in Scheme 8. The hybrid catalyst was efficiently prepared by sonicating GO with L-proline in deionized water. By this method, the native structure of L-proline was preserved so that the efficiency of this hybrid catalyst was comparable to homogeneous L-proline achieved. The efficient interlayer space of the GO carrier facilitated easy access of the substrate to active sites, resulting in an excellent yield (96%) with high ee (79%) in an aldol reaction of 2-nitrobenzaldehyde with acetone. The heterogeneous catalyst retained a highly catalytic performance in seven cycles of reuse.

In another work, Yan et al. developed a chiral BINOL-functionalized nanoporous GO catalyst (GO-BINOL-Ti) for the enantioselective addition of diethylzinc to aromatic aldehydes [40]. The catalyst GO-BINOL-Ti was synthesized by a multiple-step process, as follows: (1) commercial GO was further oxidized to produce nanoporous graphene oxide acids (GOA), in which nanopores were introduced to increase the concentration of carboxyl group, resulting in loading more BINOL molecules in the following amidation reaction; (2) (*R*)- or (*S*)-NH_2_-BINOL was synthesized from BINOL; (3) the GOA was sequentially acylated with oxalyl chloride to form GO-COCl, covalently linked to the BINOL ligands via amide bonds, and reacted with Ti(O*^i^*Pr)_4_ to generate the active catalyst GO-BINOL-Ti. The catalyst achieved good reactivity (99%) for all the screened aromatic aldehydes. The enantioselectivity was modest, and the highest ee was 45% for 1-naphthaldehyde. The low stereoselectivity was likely caused by other active sites present on GO, which might influence the stereoselectivity of BINOLs. The catalyst exhibited its recyclability over three cycles, maintaining 99% yield and 44% ee.

### 3.2. Graphene-Based Metal–Organic Complexes

**Scheme 9:**
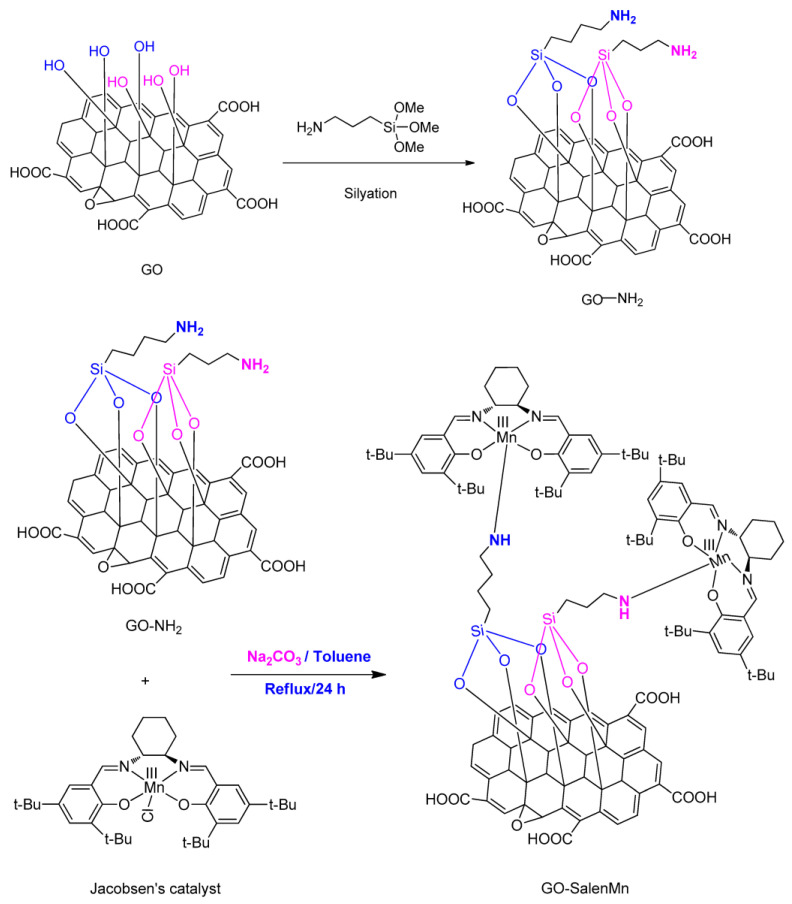
The process of GO-SalenMn synthesis. Reproduced with permission from [41].

The study by Zou et al. reported the successful synthesis of a GO-salenMn catalyst through immobilizing homogeneous chiral salenMnCl complexes onto GO modified by 3-aminopropyltrimethoxysilane (MPTMS) [41], as shown in Scheme 9. The enantioselectivity of GO-salenMn heterogeneous catalyst was greater than the salenMn homogeneous catalyst in the asymmetric epoxidation of α-methylstyrene, styrene, and indene under an m-CPBA/NMO oxidation system. The ee of the asymmetric epoxidation of α-methylstyrene increased from 52% (salenMn) to 83.2% (GO-salenMn), and the additive of NMO was necessary as the absence of NMO decreased the ee from 83.2% to 8.6%. The increase in the enantioselectivity of the heterogeneous catalyst compared to the homogeneous catalyst was attributed to the unique layered structure of GO, which provided a confinement effect enhancing chiral induction.

**Scheme 10:**
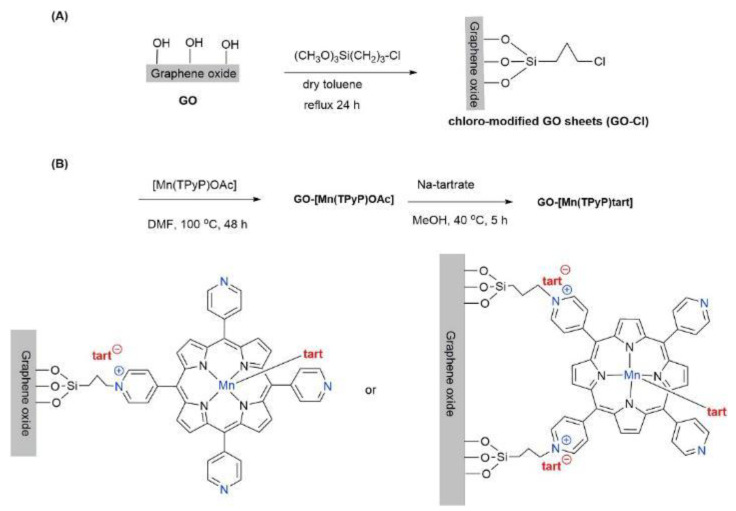
The process of GO-[Mn(TPyP)tart] synthesis: (**A**) preparation of chloro-modified GO sheets; (**B**) synthesis of the GO-[Mn(TPyP)tart]. Reproduced with permission from [42].

**Scheme 11:**
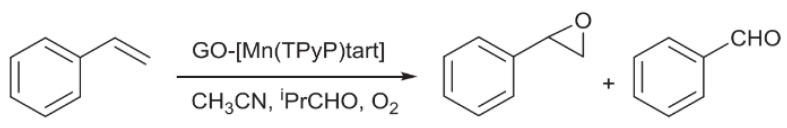
The catalysis of styrene epoxidation under O_2_/PrCHO system. Reproduced with permission from [42].

In another study, Hosseini-Monfared et al. presented a heterogeneous catalyst, GO-[Mn(TPyP)tart], which was prepared by covalently immobilizing Mn-prophyrin complex onto GO [42], as shown in Scheme 10. Similar to the study of Zou et al., the GO-[Mn(TPyP)tart] catalyst can play an important role in the asymmetric epoxidation of olefins. Full conversion with 89% epoxide selectivity and 73% ee was achieved in the catalytic epoxidation of styrene under the O_2_/PrCHO system and optimized conditions, as shown in Scheme 11. GO-[Mn(TPyP)tart] exhibited great recyclability with the retained yield and enantioselectivity over five cycles.

Hosseini-Monfared et al. introduced a heterogeneous chiral imino indanol complex of manganese ([Mn(L)(OH)]) immobilized on carbon-coated magnetic Fe_3_O_4_ nanoparticle-decorated reduced graphene oxide sheets (GFC) [43].The catalyst exhibited exceptional efficiency in the aerobic epoxidation of olefins using O_2_ and isobutyraldehyde, achieving up to 98% conversion and 100% enantioselectivity. The graphene support enhanced electron density around the Mn center and also provided a high surface area for reactant adsorption. Magnetic Fe_3_O_4_ nanoparticles facilitated easy recovery, with the catalyst retaining reactivity over five cycles. The rigid indanol unit of the ligand was critical in high enantioselectivity because of its high asymmetric induction.

Schulz et al. reported a new strategy for the immobilization of metal–salen complex onto rGO via π-π noncovalent interactions [44]. They designed a salen ligand functionalized with pyrene groups, which was immobilized onto the rGO surface by noncovalent interactions and retained the activity of the parent Cr-salen complex. In the presence of this catalyst, high catalytic activity and enantioselectivity in asymmetric reactions, such as the hetero-Diels–Alder reaction and asymmetric ring-opening of meso epoxide were obtained. Moreover, this catalyst has exceptional recyclability, retaining 96% conversion and 68% ee after 10 cycles in the asymmetric ring-opening of cyclohexane oxide.

In a similar approach, Shi et al. presented a hybrid catalyst composed of multicomponent chiral catalyst (MCC) modified with the pyrene group [45]. The MCC immobilized onto graphene via the π-π stacking interactions between the pyrene group and graphene. This “in situ immobilization” preserved the catalyst’s well-defined structure, and the structure of the MonoPhos/Rh^I^ catalyst was maintained during the immobilization, enabling the asymmetric hydrogenation of dehydroamino acid esters completed with high yield and enantioselectivity.

**Scheme 12:**
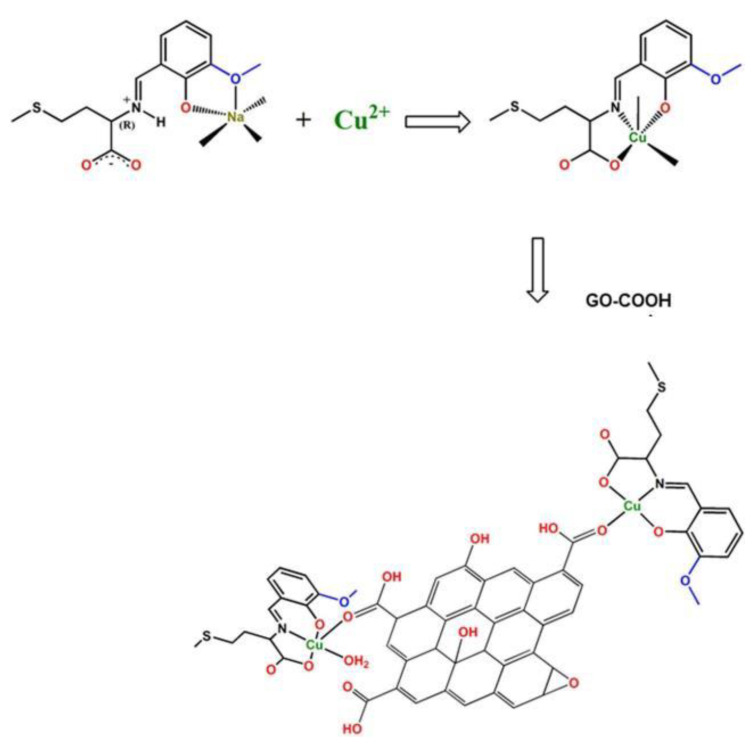
The process of GO-supported Cu-valmet complex synthesis. Reproduced with permission from [46].

**Scheme 13:**
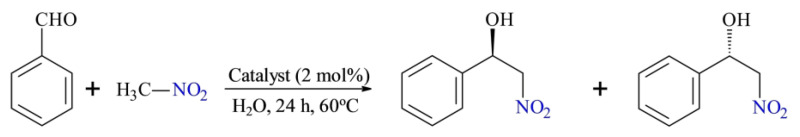
Henry reaction of benzaldehyde and nitromethane. Reproduced with permission from [46].

Pârvulescu et al. immobilized the L- or D-valmet copper(II) complex on GO as a heterogeneous catalyst, as shown in Scheme 12. By anchoring the copper(II) complexes onto carboxyl-modified GO [46], the obtained catalyst exhibited excellent performance in Henry, cyanosilylation, and aldol reactions. As shown in Scheme 13, the Henry reaction product was obtained with 92.5% conversion and 95.8% ee by the catalysis of the GO-supported Cu-valmet complex.

## 4. Other Carbon Materials Based Asymmetric Catalysts

In addition to the materials mentioned above, researchers have explored other carbon materials as catalyst supports, such as nanodiamonds [47,48], active carbon [49,50], fullerene [51,52,53], and chiral carbon dots [54,55,56,57,58,59,60]. These materials also exhibited unique properties in asymmetric catalysis.

Nanodiamonds is a kind of nanoscale carbon particle with diamond lattice. Due to its high surface area, tunable surface structures, and non-toxic properties, this material has emerged as a promising candidate for a wide range of applications in the fields of organic and biomedical research [47].

Chen et al. explored a method for chemically modified ultra-dispersed nanodiamonds to introduce functional groups that enhance their applicability in various fields, including catalysis [48]. The unique aspect of this work lies in that they converted surficial hydroxyl groups of nanodiamonds into functionalizable moieties, such as oxyhexanol groups. This modification allows for the further covalent attachment of various functional moieties, including amine, cyanide, azide, and thiol, thereby expanding the potential applications of nanodiamonds. In terms of catalysis, the functionalized nanodiamonds were explored as a new catalyst support for peptide synthesis. The study demonstrated that nanodiamonds modified with oxyhexanol groups could effectively anchor chiral ligands, thus forming heterogeneous catalysts. Specifically, the covalent attachment of chiral proline ligands to nanodiamonds yielded a new enantioselective catalyst for the asymmetric aldol reaction. Although this catalyst exhibited moderate enantioselectivity under solvent-free conditions, nanodiamonds proved to be a qualified support for asymmetric catalysis.

**Scheme 14:**
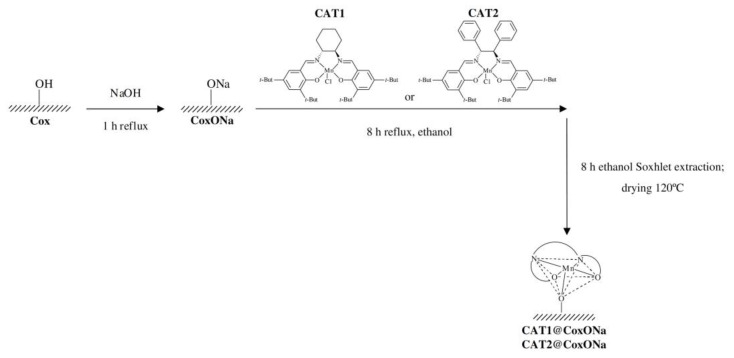
The process of CAT1@CoxONa and CAT2@CoxONa synthesis. Reproduced with permission from [50].

In another two studies, researchers reported the successful anchoring of chiral manganese(III) salen complexes onto activated carbon using a straightforward axial coordination method for the synthesis of CAT1@CoxONa and CAT2@CoxONa, as shown in Scheme 14 [49,50]. The method is featured with the effective immobilization of salen complexes, which showed high enantioselectivity in homogeneous catalysis, onto a cost-effective and porous support material. This immobilization process enhances the recyclability and economic viability of the catalysts, while maintaining significant catalytic activity. The active carbon-based catalysts demonstrated enantioselective activity in the epoxidation of styrene, with comparable or even improved enantioselectivity compared to homogeneous counterparts, especially when using NaOCl as the oxidant. Notably, the catalysts showed minimal metal leaching and maintained activity over multiple cycles, highlighting the robustness of the immobilization method.

Chiral carbon quantum dots (cCQD), an emerging class of fluorescent carbon nanomaterials (<10 nm), have recently transcended their traditional roles in sensing and bioimaging to become versatile platforms for asymmetric catalysis [54,55]. Their tunable surface chemistry, biocompatibility, and capacity to retain molecular chirality from chiral precursors enable efficient asymmetric control in reactions such as aldol condensations and Michael additions. The key advancements in cCQD-based asymmetric catalysts are summarized in the following paragraphs, emphasizing their design strategies, catalytic mechanisms, and performance.

**Figure 5:**
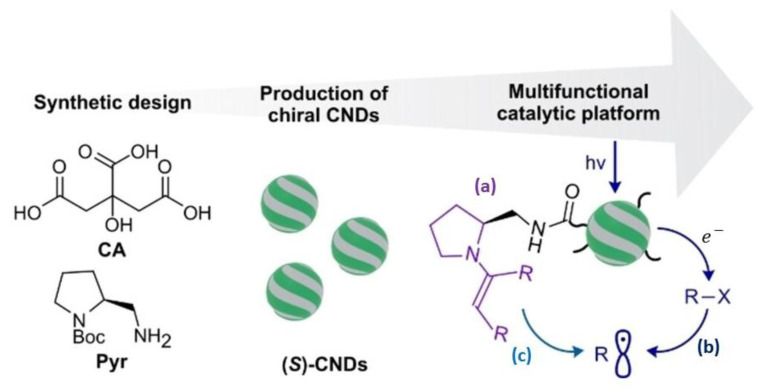
The scheme of the synthesis of (*S*)-CNDs and their multiple catalytic uses in (a) organocatalytic, (b) photoredox and (c) organophotoredox processes. Reproduced with permission from [57].

As a chiral catalyst support, cCQDs with various structures have been synthesized and used to achieve good performance in aldol reactions [56,57,58,59,60]. Prato et al. pioneered cCQD-based asymmetric catalysis by immobilizing amine-rich structures on the CQD surface [56]. By characterizing the types and quantities of surficial amines in detail, they have employed this type of novel covalent organic catalysts in aqueous media with high performances. These carbon dots can efficiently catalyze the amine-catalyzed transformation of carbonyl compounds through both iminium ion and enamine activation pathways, including conjugate addition and aldol reactions, exhibiting high yields and certain asymmetric selectivity (ee = 38%). Asymmetric catalysis can be achieved in the presence of cCQDs, providing new insights into the application of carbon-based materials in aqueous-phase organic catalysis. Subsequently, this group prepared a new type of cCQDs via microwave-assisted synthesis followed by simple purification [57], as shown in Figure 5. These cCQDs exhibited excellent catalytic performance in organic and photochemical reactions, facilitating various chemical transformations. The composition of their surface functional groups allows them to show high asymmetric selectivity for aldol reactions (ee > 90%) and to drive reactions through photochemical processes, even promoting cross-dehydrogenative coupling reactions by simultaneously utilizing photoredox and organocatalytic activation. The material’s photochemical properties have been successfully exploited to compensate for the shortcomings of traditional molecular catalysts.

**Figure 6:**
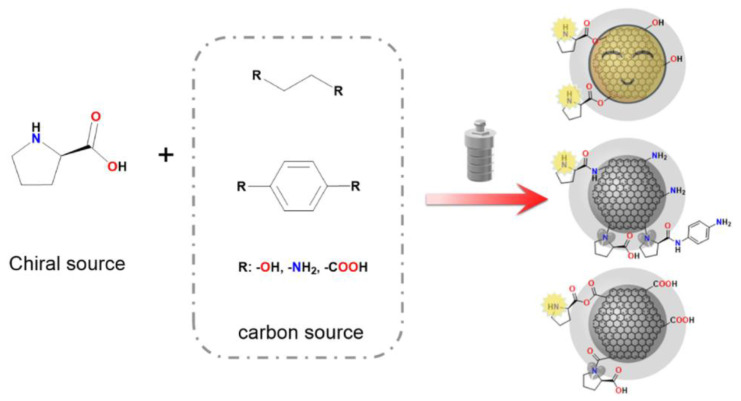
The scheme of the synthesis of chiral CDs using different carbon sources. Reproduced with permission from [59].

Xu et al. have also achieved many results in the field of cCQD-based asymmetric catalysis [58,59]. They synthesized cCQDs via a one-step hydrothermal method, using citric acid and D-proline as precursors and conducting the reaction for 2 h at 180 °C [58]. The obtained carbon dots exhibited high catalytic performance in direct aldol addition between *p*-nitrobenzaldehyde and cyclohexanone with a yield of 98% and an ee value of 73%. In addition, they conducted an in-depth study on the relationship between the structure of carbon sources and catalytic performance [59]. The authors found that the skeletal structure (conjugated or non-conjugated) and functional groups (hydroxyl, carboxyl, and amino groups) of the carbon sources significantly influence the asymmetric catalytic activity and reproducibility of carbon dots, as shown in Figure 6. The results show that hydroxyl functional groups in the carbon source are more beneficial to enhancing the catalytic activity and reproducibility of carbon dots, providing an effective method for the controlled synthesis of carbon dots with high asymmetric catalytic performance.

**Figure 7:**
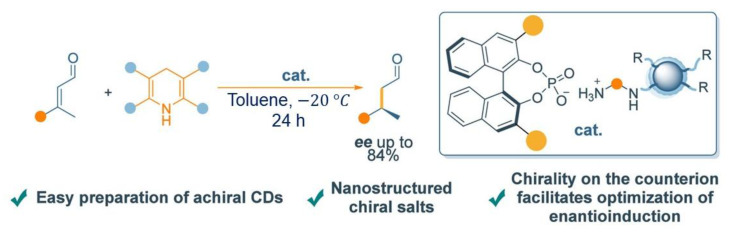
The scheme of the asymmetric catalytic use of achiral CDs. Reproduced with permission from [63].

In addition, cCQDs have shown good application prospects in asymmetric oxidation, reduction, and other reactions [61,62,63]. Kand et al. proposed a method for preparing cCQDs with enantioselective catalytic activity through the electrochemical oxidative polymerization of serine enantiomers [61]. With the sizes of 2–7 nm, these CQDs possess a well-defined polycyclic dipeptide core structure and a hexagonal symmetric spatial structure, exhibiting enantioselective catalytic activity toward the oxidation of 3,4-dihydroxyphenylalanine (DOPA). Ramazani et al. studied a chiral pseudohomogeneous catalyst (PHC) based on amphiphilic CQD for controlling the enantioselectivity of the Kharasch−Sosnovsky reaction [62]. This PHC combines the advantages of homogeneous and heterogeneous catalysts, demonstrating excellent catalytic performance. Under optimized conditions, a 95% conversion rate and ee value of 75% were achieved. It was found that chiral information can be effectively transferred from the shell of the nanocatalyst, thereby inducing enantioselectivity in the C-H activation and subsequent C-O formation. In addition, the catalyst is easily recovered from the reaction medium, showing good potential for practical application. Carlone et al. proposed an application of nitrogen-doped carbon dots in asymmetric iminium ion catalysis through asymmetric counterion-directed catalysis [63]. The achiral CQDs combined with chiral phosphoric acid can achieve an asymmetric catalytic reduction in α,β-unsaturated aldehydes, as shown in Figure 7. By optimizing the reaction conditions, it was found that their catalytic performance was superior to both other tested CQDs and t simple molecular amines, exhibiting higher TON and TOF. In addition, the formation of iminium ions on the CQDs was confirmed by NMR spectroscopy. This work not only demonstrates the potential of CQDs in asymmetric nanocatalysis but also emphasizes the importance of CQDs as organic catalysts in the field of materials science, providing new insights for the future application of CQDs in other catalytic processes.

## 5. Conclusions

In recent years, carbon-based materials have exhibited great application potential in heterogeneous asymmetric catalysis as excellent supports. Their unique structures and chemical properties allow various types of anchoring methods, such as covalent and noncovalent approaches, which have expanded the strategies available for diverse functionalization. Accordingly, carbon materials exhibit broad applicability in various types of asymmetric reactions due to their high compatibility with chiral catalysts. Moreover, the immobilization of a homogeneous chiral catalyst onto carbon materials, as a green and sustainable strategy, can minimize the use of toxic solvents and improve the separation and reusability of the chiral catalyst. Thus, the high catalytic activity, selectivity, and recyclability of heterogeneous carbon-based asymmetric catalysts tends to offer more significant advantages over the corresponding traditional homogeneous counterparts, especially in terms of reusability. Nevertheless, despite their excellent catalytic properties, their practical deployment on a large scale is still constrained by certain problems.

Looking ahead, there are still several challenges to overcome. First, the scalable production of carbon-based chiral catalysts remains a major challenge. Current synthetic methods are often complex, involving multiple synthetic steps, which makes many of these strategies time consuming, costly, and not environmentally friendly. These limitations inhibit their practical industrial applications. Second, the chirality of most carbon-based chiral catalysts is unstable, which leads to a reduction in their reusability after several catalytic cycles. To address the challenges associated with the scalable production, there are some possible solutions, such as the use of suitable chiral ligands, templating techniques, and supramolecular self-assembly, as highlighted in the aforementioned examples. Furthermore, machine learning (ML) and artificial intelligence (AI) are expected to play a crucial role in accelerating the discovery and design of chiral catalysts. Some studies have already employed ML to predict the performance of heterogeneous catalysts and evaluate catalyst design strategies [64,65]. Similarly, emerging research has begun to apply ML to chiral catalyst systems, aiming to address key challenges, such as enantioselectivity prediction [66,67,68]. These predictive models can accelerate the discovery and optimization of chiral catalysts by enabling the rapid screening of ligand structures and reaction conditions.
